# Midterm Outcome of AB0 Incompatible Kidney Transplantation in Children and Adolescents—A Single Center Experience

**DOI:** 10.1111/petr.70248

**Published:** 2026-01-22

**Authors:** Christina Taylan, Sabine I. Mückenhausen, Lutz T. Weber, Dirk L. Stippel, Julia Thumfart

**Affiliations:** ^1^ Pediatric Nephrology, Children's and Adolescents' Hospital University Hospital of Cologne, Faculty of Medicine, University of Cologne Cologne Germany; ^2^ Department of General‐, Visceral‐, Thoracic‐ and Transplantation Surgery University of Cologne, Faculty of Medicine and University Hospital of Cologne, University Hospital of Cologne Cologne Germany; ^3^ Department of Pediatric Gastroenterology, Nephrology and Metabolic Diseases Charité Universitätsmedizin Berlin Berlin Germany

**Keywords:** graft loss, immunoadsorption, kidney transplant, plasma exchange

## Abstract

**Background:**

In order to reduce the waiting time, new strategies have been developed to safely transplant donor and recipient pairs with mismatched blood groups. The present study examined the safety of the preparatory treatments and the midterm outcome of AB0 incompatible (AB0i) kidney transplantation in children.

**Methods:**

We retrospectively analyzed 10 children who received a kidney transplant from an AB0i donor from 2012 to 2024 and 30 patients matched by sex, height, and weight who received an AB0 compatible (AB0c) living kidney transplant in the same period.

**Results:**

In the AB0i group, preparatory treatment before KTx was well tolerated. The number of rejection episodes was comparable in the AB0i group and in the AB0c group (three episodes in three patients vs. seven episodes in six patients) during the observation period of 36 months, with rejections occurring earlier in the AB0c group. Infections were more frequent in the AB0c group than in the AB0i group (30 episodes in 23 patients [76%] vs. eight episodes in six patients [60%]). In the AB0i group, the 3‐year graft survival rate was 90%; the 3‐year patient survival was 100%. In the AB0c group, the 3‐year graft survival was 86.8%, and the 3‐year patient survival was 93.3%. There was no difference in graft survival (*p* = 0.08) and patient survival (*p* = 0.24) between the AB0i and AB0c groups.

**Conclusions:**

AB0 incompatible kidney transplants can be safely performed in children with equivalent midterm graft and patient survival.

## Graphical abstract

AB0‐incompatible kidney transplantation in children can be performed with excellent results. Midterm outcome of AB0 incompatible KTx was not inferior to that of AB0 compatible KTx. Graft survival and graft function were comparably good in both groups. There was no increase in infectious complications or malignancies with AB0i KTx.

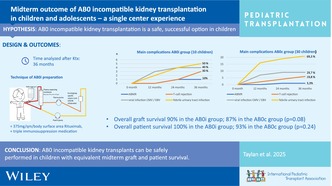

**Abbreviations**
AB0c: AB0 compatible
AB0i: AB0 incompatible
ABMR: antibody mediated rejection
BSA: body surface area
BW: body weight
CAKUT: congenital anomalies of kidney and urinary tract
CKD: chronic kidney disease
CMV: cytomegalovirus
CsA: cyclosporine A
DSA: donor specific antibodies
EBV: Epstein–Barr virus
eGFR: estimated glomerular filtration rate
FFP: fresh frozen plasma
IA: immunoadsorption
Ig: immunoglobulin
Ktx: kidney transplantation
MMF: mycophenolate mofetil
PE: plasma exchange
PTLD: posttransplant lymphoproliferative disease
Tac: tacrolimus

## 1 Introduction

In pediatric chronic kidney disease (CKD), kidney transplantation (KTx) represents the optimal treatment option. There is a notable discrepancy between the number of donor organs offered and the number of potential recipients that varies among different societies and countries, resulting in significant differences in waiting time. In Germany, the waiting period for children under the age of 18 is approximately 3–4 years [1]. In the U.S., the waiting time varies depending on the state in which the child is listed and is about 12 months nationwide [2].

Survival benefits have been shown for transplanted children compared to children on the waiting list on dialysis [3]. The time spent on dialysis is highly relevant for children, as both physical and psychosocial development are significantly impaired during the time on dialysis [4].

Children with CKD are exposed to many cardiovascular risk factors (e.g., anemia, metabolic acidosis, hyperphosphatemia, hyperparathyroidism, and dyslipidemia). It has been shown that cardiovascular risk, once established in childhood, is inexorably progressive and can cause progressive damage into adulthood. Early transplantation with the shortest possible duration of the above risk factors and avoidance of severe diastolic blood pressure fluctuations, as often occur with dialysis, seems to have a positive effect on the cardiovascular risk of these children [5, 6, 7]. Besides the encouragement for living donation other innovative strategies are necessary to expand the donor pool, namely crossover KTx and acceptance of non‐heart‐beating donors, both of which are legal only in some countries [8, 9]. Another approach is offered by living donor KTx despite AB0 incompatibility (AB0i) between donor and recipient [10, 11]. AB0 isoagglutinins are predominantly immunoglobulin (Ig)M antibodies, although IgG and IgA classes also exist in plasma. The AB0 blood group antigens are expressed by the endothelium of solid organs. Isoagglutinins are produced in response to the presence of foreign antigens from approximately 6 months of age [12]. The organism develops antibodies against the antigenic properties A and/or B under immune tolerance of its own blood group. All AB0 isoagglutinins can activate the complement system and cause intravascular lysis [13]. The contact of blood group antigens and isoagglutinins of different blood groups leads to hyperacute antibody‐mediated allograft rejection. Consequently, AB0i has historically constituted the primary impediment to KTx.

Strategies to remove existing antibodies and prevent new antibody formation permit AB0i KTx. These procedures have been established in Belgium and Japan in the 1980s and have become routine worldwide by the mid‐2000s. While there is encouraging and extensive data for adult KTx [14], there is still a paucity of information on pediatric protocols and outcomes [15].

This study presents the midterm outcome over 36 months after KTx for a pediatric AB0i KTx cohort in comparison to a matched group of pediatric AB0 compatible (AB0c) KTx at our center from 2012 to 2024.

## 2 Patients and Methods

### 2.1 Patient Cohort

From January 2012 to March 2024, 10 AB0i KTx were performed at the Pediatric Transplant Center of the University Hospital of Cologne. AB0i KTx was defined by blood group incompatibility of the recipient with the donor (e.g., blood group A or B or AB donor to blood group 0 recipient, or blood group A or AB donor to blood group B recipient, or blood group B or AB donor to blood group A recipient). The Pediatric Transplant Center of the University Hospital of Cologne prioritizes living kidney donation, and when blood group‐identical related donors are not available, ABOi living related KTx is considered. All AB0i KTx between January 2012 and March 2024 were included in this analysis. This was a purely retrospective, anonymized data analysis that had been submitted to the Ethics Committee of the Medical Faculty of Cologne (file number: 25‐TEMP204966‐retro) before analysis.

Patient and allograft survival were compared between the AB0i and a group of 30 AB0c living‐related KTx recipients matched for sex, age, and weight who underwent transplantation at our center during the same period.

We compared patient and graft survival and the incidence of acute rejection and infection at 1, 3, 6, 12, 24, and 36 months after KTx.

The primary renal diagnosis was categorized as CAKUT, glomerulopathies, cystic kidney disease, and others. Demographic data including sex, age, body weight, height, body mass index, time on dialysis, HLA mismatch, panel reactive antibody, and blood group isoagglutinin titer were extracted from the medical records just prior to treatment. Immunosuppressive therapy was assessed from medical records 4 weeks after KTx.

Estimated glomerular filtration rate (eGFR) was calculated using the Full Aged Spectrum formula [16].

### 2.2 Preparatory Treatment of the AB0i Recipients

All blood group combinations were classified as AB0i if they were not the same blood group or if the donor did not have blood group 0 or the recipient did not have blood group AB. One patient with the A2B combination was also transplanted after prior preparation. All AB0i recipients received a single dose of rituximab (375 mg/m^2^ body surface area) 38.5 (±4.7) days prior to scheduled transplantation. Eight to ten days prior to the planned KTx (depending on the isoagglutinin titer), seven of ten patients were pretreated with immunoadsorption (IA) performed with apheresis devices (SigmaPlasauto, Diamed Medizintechnik, Cologne, Germany), hollow‐fiber plasma separators (Plasmaflo OP 02 or 05 AsahiKasei Medical Japan), and an antibody adsorber (Glycosorb ABO, Glycorex Sweden). In three infants with low anti‐A/anti‐B titers < 1:1 initially, no antigen‐specific IA was performed. In all apheresis sessions, unfractionated heparin was used for anticoagulation.

The goal was to achieve an isoagglutinin titer ≤ 1:4 for IgM and IgG the day before KTx. If this could not be achieved, plasma exchange (PE) was also performed. In *n* = 4 patients isoagglutinin titers < 1:4 could not be reached with IA alone, so 1–6 sessions of PE were applied in addition. PE was performed with SigmaPlasauto devices (Diamed Medizintechnik, Cologne, Germany) and hollow‐fiber plasma separators (Plasmaflo OP 02 or 05 AsahiKasei Medical Japan).

The type of substitution solution, either fresh frozen plasma (FFP) or human albumin 5%, depended on the individual blood coagulation status; ≤ 2 days before KTx a fibrinogen concentration > 2 g/L was aimed for.

The total number of therapeutic apheresis sessions performed depended on the initial titer, the drop in titer after treatment, and rebound before the next treatment.

After KTx, isoagglutinin titers were monitored daily in the first 2 weeks. If titers exceeded 1:8 IgM/IgG in the first week or 1:16 IgM/IgG in the second week, IA was performed.

### 2.3 Isoagglutinin Titer Measurements, Cross‐Matching and Alloantibody Detection

The quantitative determination of the isoagglutinin titer was performed using an agglutination test in which an antigen–antibody reaction is performed in a dilution series. At our center, the immunohematology laboratory uses a gel card test from Bio‐Rad Laboratories GmbH. IgM was determined by sodium chloride cards and IgG by Coombs cards. The indirect Coombs test was performed using donor erythrocytes or pooled erythrocytes.

### 2.4 Immunosuppression and Induction

Five days prior to KTx, oral immunosuppression was initiated with tacrolimus (Tac) in children over 5 years of age (0.2 mg/kg/day in children > 40 kg and 0.3 mg/kg bodyweight (BW)/day in children < 40 kg) or cyclosporine A (CsA) in children below 5 years of age (300 mg/m^2^ body surface area (BSA)/day) and mycophenolate mofetil (MMF) (in combination with Tac 1200 mg/m^2^ BSA/day, in combination with CsA 1800 mg/m^2^ BSA/day). MMF was reduced by a quarter in combination with Tac and reduced by a third in combination with CsA 2 weeks after KTx. In the first 4 weeks after kidney transplantation, target trough levels were 8–12 ug/L for tacrolimus and 140–200 μg/L for cyclosporine. There was no difference in target levels for AB0i and AB0c transplanted children. In all patients of the AB0i group, basiliximab induction (10 mg in children < 35 kg BW and 20 mg in children > 35 kg BW) was given at KTx and on postoperative day 4. In the AB0c group, basiliximab was given when patients qualified (< 15 kg BW, known poor medication adherence in the past, second KTx). Prednisone (P) was started at 360 mg/m^2^ BSA intravenously at surgery, followed by 60 mg/m^2^ BSA orally on day 1, 38 mg/m^2^ BSA on day 2 postoperatively and then tapered to 4 mg/m^2^ BSA on postoperative day 43.

### 2.5 Anti‐Infective Prophylaxis in AB0‐Incompatible and AB0‐Compatible Recipients

Preoperatively, all recipients received a single dose of 50 mg/kg BW cephazolin intravenously (i.v.). All recipients received antibacterial prophylaxis against Pneumocystis infection with trimethoprim‐sulfamethoxazol for 6 months.

A cytomegalovirus (CMV) prophylaxis with valganciclovir was given to all recipients except for those with a donor negative/recipient negative anti‐CMV‐IgG constellation for the first 100 days after KTx. The valganciclovir dose was adjusted based on BSA and graft function.

An Epstein Barr virus (EBV) prophylaxis was given to all anti‐EBV‐IgG negative recipients, who received a graft of an anti‐EBV‐IgG positive donor or a donor with unknown anti‐EBV status. The recipients who qualified for EBV prophylaxis received CMV hyperimmunoglobulin within the first 72 h post KTx and 2, 4, 6, and 8 weeks post KTx in a dose of 100 mg/kg BW i.v., as well as 50 mg/kg BW i.v. in weeks 12 and 16 post KTx in addition to valganciclovir prophylaxis for the first 100 days after KTx [17]. After transplantation EBV, CMV, and BKV PCR were performed every 3 months. The virus status of all recipients and donors prior to transplantation is listed in the supplement table.

### 2.6 Data Analysis and Presentation

This is a retrospective comparative study based on patient characteristics taken from clinical records.

Infectious complications with elevated infection parameters (C‐reactive protein, leukocytosis, leukocyturia) were divided into febrile infections and sepsis if the Phoenix sepsis score permitted a corresponding classification. CMV and EBV disease were defined by virus replication with clinical symptoms. When the children suffered from diarrhea and vomiting, gastrointestinal infections were detected by stool virological or bacterial analysis.

Noninfectious complications included posttransplant lymphoproliferative disease and surgical complications. Graft failure was defined by the need to resume dialysis permanently. Delayed graft function was defined as the need for at least one dialysis treatment within the first week after KTx. Transplant kidney biopsies were performed at the time of KTx and whenever indicated by creatinine increase > 10% after ruling out other causes like infections and prerenal causes.

### 2.7 Statistical Analysis

Normality was tested by the Shapiro–Wilk test. Not normally distributed data are expressed as median and range. If data are normally distributed, it is expressed as mean and standard deviation. Group comparison was performed with the *t*‐test for normally distributed data and the Mann–Whitney Rank sum test for not normally distributed data. SigmaPlot for Windows Version 16 (graffiti, wpcubed GmbH, Germany) software was used for statistical calculation. Statistical significance was assumed at a *p* value of < 0.05.

## 3 Results

### 3.1 Patient Characteristics and Preparatory Treatment

We analyzed 40 patients with a median age of 12 years (range 2–17 years) at the time of transplantation who underwent KTx between 2012 and 2024. Of these, 10 transplants were AB0 incompatible; each of the AB0i KTx was analyzed as matched with three AB0 compatible transplanted children.

Primary renal diagnoses and medical characteristics of the study cohort are shown in Table 1. Patient characteristics of AB0i transplants are analyzed in more detail, including blood group mismatch, anti‐A/B titers, number of IA/PE sessions required, and relationship to donor (Table 2). Immunological risk factors in both groups are given in Table 3.

**TABLE 1:** Characteristics of the study cohort before KTx.

	AB0i KTx (*n* = 10)	AB0c KTx (*n* = 30)	*p*
Age (years)	10.3 ± 7.5	10.2 ± 6.1	0.49
Body weight (kg)	31.1 ± 20.3	32.5 ± 18.5	0.57
Height (cm)	128.3 ± 37.7	127.6 ± 35.7	0.59
BMI (kg/m^2^)	16.8 ± 2.20	17.8 ± 2.9	0.88
Sex male/female	7 (70%)/3 (30%)	18 (60%)/12 (40%)	
Primary renal diagnosis			
CAKUT	4 (40%)	13 (43.3%)	0.65
Glomerulopathy	2 (20%)	9 (30%)	
Cystic kidney disease	3 (30%)	3 (10%)	
Other^a^	1 (10%)	5 (16.6%)	
Time on dialysis before KTx (months)	3 (range 0–60)	8 (range 0–29)	0.85
Preemptive KTx	4 (40%)	11 (36.6%)	0.91

*Note:* Data are presented as mean ± SD or as numbers (%).

Abbreviations: AB0c, AB0 compatible; AB0i, AB0 incompatible; BMI, body mass index; CAKUT, congenital anomalies of the kidney and urinary tract; KTx, kidney transplantation.

^a^ Other diagnoses are: feto‐fetal transfusion syndrome, perinatal asphyxia, Bartter type IV syndrome, meningomyelocele with neurogenic bladder disease, bilateral renal vein thrombosis.

**TABLE 2:** Patient characteristics of the AB0 incompatible cohort.

Sex	Age at KTx (years)	AB0	Initial Anti‐A/B IgG/IgM titer	Number of IA/PE	Anti‐A/B IgG/IgM titer before KTx	Donor	Primary renal diagnosis
Male	2	A1‐B	< 1:1/< 1:1	0	< 1:1/< 1:1	Father	Urethral valve
Male	3	A1‐0	1:128/1:8	8/1	1:2/< 1:1	Mother	CAKUT, Dysplasia
Male	21	A2‐0	1:16/1:32	6/0	1:4/1:1	Father	Single kidney, sp. ALL
Female	2	A2‐0	< 1:1/< 1:1	0	< 1:1/< 1:1	Father	CAKUT, Dysplasia
Female	16	B‐0	1:64/1:64	6/1	1:4/1:4	Father	Denys–Drash‐Syndrom, WT 1 mutation
Male	13	A2‐B	1:8/1:8	6/0	< 1:1/< 1:1	Mother	Congenital nephrotic syndrome
Male	2	B‐A	< 1:1/1:4	0	< 1:1/< 1:1	Father	HNF1 Beta Nephropathy
Female	11	B‐0	1:256/1:128	7/1	1:4/1:4	Father	Nephronophtisis (NPHP1‐Mutation)
Female	13	B‐0	1:16/1:4	4/0	< 1:1/< 1:1	Father	Joubert Syndrome (NPHP6‐Mutation)
Male	21	A1‐0	1:256/1:64	4/6	1:4/1:1	Father	Feto‐fetal transfusion syndrome, perinatal multi organ failure

Abbreviations: IA, immunoadsorption; IgG, immunoglobulin G; IgM, immunoglobulin M; PE, plasma exchange; sp. ALL, state post‐acute lymphatic leukemia.

**TABLE 3:** Immunological risk factors of all patients.

Immunological risk factors		AB0i KTx (*n* = 10)	AB0c KTx (*n* = 30)
HLA mismatch			
A	0	2 (20%)	13 (43.3%)
	1	8 (80%)	17 (56.6%)
	2	0	0
B	0	0	19 (63.2%)
	1	10 (100%)	10 (33.3%)
	2	0	1 (3.33%)
DR	0	4 (40%)	8 (26.6%)
	1	5 (50%)	19 (63.2%)
	2	1 (10%)	3 (9.99%)
DQ	0	6 (60%)	13 (43.3%)
	1	4 (40%)	16 (53.3%)
	2	0	1
No of KTx			
First		9	29
Second		1	1
Panel reactive antibodies			
0%–19%		100%	100%
20%–79%		0%	0%
> 80%		0%	0%

*Note:* Data are given as number (%).

Abbreviations: AB0c KTx, AB0 compatible kidney transplantation; AB0i KTx, AB0 incompatible kidney transplantation.

A total of 43 IA and 9 PE sessions were performed. The median number of IA per patient (*n* = 7) was 6 (range 4–8) and 1 (range 0–6) for PE (*n* = 4). There were no problems with vascular access or tolerability of therapeutic apheresis during any session.

### 3.2 Immunosuppressive Therapy

We found a higher specific immunosuppressive load in the AB0i group, by calculating the pediatric modified Vasudev score [18]. Four weeks after KTx, the median modified Vasudev score in the group of AB0i transplanted children was 17.3 (8.09–14.4), while the group of AB0c transplanted children had a statistically significant (*p* < 0.001) lower median score of 11.0 (13.3–28.3).

Four weeks after KTx, the median trough level of CsA in the AB0c group was 148 (126–198) μg/L. In the AB0i group, only one child received CsA with a trough level of 140 μg/L 4 weeks after KTx. For Tac, results at week 4 after KTx were 10.6 (5.5–15.6) μg/L in the AB0c group and 12 (10.7–13.5) μg/L in the AB0i group. The median dose of cyclosporine in the AB0c group was 172.3 (150.9–244.9) mg/m^2^ BSA/day 4 weeks after KTx. In the AB0i group, the dose of the single child was 188.6 mg/m^2^ BSA/day 4 weeks after KTx. The median tacrolimus dose in the AB0c group 4 weeks after KTx was 3.9 (2–9) mg/m^2^ BSA/day, while the dose in the AB0i group was 11.9 (2.3–14.9) mg/m^2^ BSA/day. This reflects a statistically significant higher dose for both calcineurin inhibitors in the AB0i group (*p* = 0.001).

### 3.3 Kidney Function After Transplantation

**FIGURE 1:**
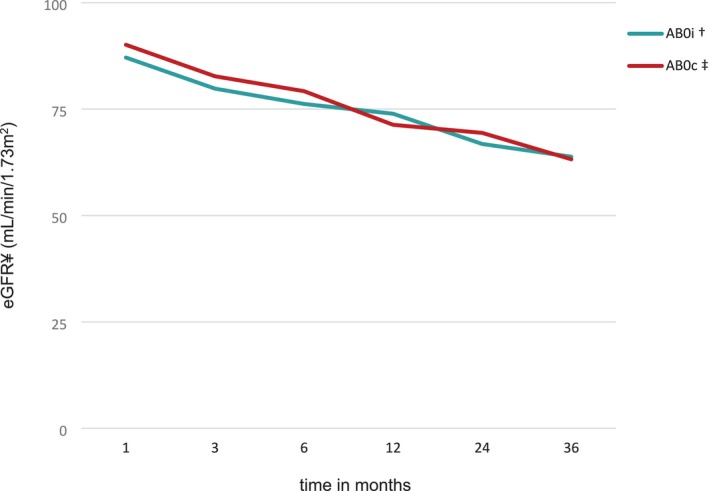
Allograft function after AB0c and AB0i KTx. Median eGFR using the full aged spectrum formula is shown over 36 months. ¥eGFR estimated glomerular filtration rate, ^†^AB0i AB0 incompatible transplantation, ^‡^AB0c AB0 compatible transplantation.

In the AB0i KTx group, 80% (8 of 10) of the children analyzed were available for a follow‐up at 36 months. In the AB0c KTx group, 80% (24 of 30) of the patients were available for a follow‐up visit at 36 months. Figure 1 depicts the eGFR in mL/min/1.73 m^2^ BSA during the first 36 months after KTx. Comparing the results of the 10 AB0i KTx patients with those of the 30 AB0c children, we found no statistical difference in GFR between the AB0i and the AB0c group of up to 3 years of follow‐up.

### 3.4 Main Complications After Transplantation

All 40 children were prepared and transplanted according to the above‐mentioned protocol and according to their group affiliation (AB0i KTx or AB0c KTx).

The overall incidences of complications were 34% within the first year, 33% within the second year, and 24% during the third year after transplantation (see Tables 4 and 5). Occurrence of complications was comparable in the two groups.

**TABLE 4:** Complications within the first 36 months in AB0i KTx group.

	12 months (*n* = 10)	24 months (*n* = 9)	36 months (*n* = 8)
ABMR	0	1	0
T‐cell rejection	0	1	1
CMV infection	1	0	0
EBV infection	2 in 1 patients	1	0
PTLD	0	0	0
Urosepsis	1	0	0
Febrile urinary tract infections	1	2 in 1 patient	1
BKV nephropathy	0	0	0

*Note:* EBV/CMV infections are to be understood as a disease. Asymptomatic viremia is not included in the table. All febrile infections or infections underlying sepsis were infections of the urinary tract. A complication was classified as sepsis if the Phoenix sepsis score permitted a corresponding classification.

Abbreviations: ABMR, antibody mediated rejection; CMV, cytomegalovirus; EBV, Epstein Barr virus; PTLD, posttransplant lymphoproliferative disease.

**TABLE 5:** Complications within the first 36 months in AB0c KTx group.

	12 months (*n* = 30)	24 months (*n* = 27)	36 months (*n* = 25)
ABMR	0	1	0
T‐cell rejection	2 in 2 patients	2 in 2 patients	2 in 1 patient
CMV infection	3 in 3 patients	1 in 1 patients	0
EBV infection	4 in 4 patients	1	0
PTLD	2 in 2 patients	0	0
Urosepsis	2 in 2 patients	2 in 1 patient	1
Febrile urinary tract infections	14 in 9 patients	1	1
BKV nephropathy	3 in 2 patients	0	0

*Note:* EBV/CMV infections are to be understood as a disease. Asymptomatic viremia is not included in the table. All febrile infections or infections underlying sepsis were infections of the urinary tract. A complication was classified as sepsis if the Phoenix sepsis score permitted a corresponding classification.

Abbreviations: ABMR, antibody mediated rejection; CMV, cytomegalovirus; EBV, Epstein Barr virus; PTLD, posttransplant lymphoproliferative disease.

All patients had a functioning graft directly after surgery. In all but one of the 40 patients, the postoperative course was remarkably uneventful.

This one patient of the AB0c group had urosepsis immediately after transplantation and required one single dialysis session on day 12 post KTx due to massive fluid overload. From day 28 post KTx, the patient had normalized kidney function. Overall, infectious complications within the first 90 days following KTx were lower in the AB0i cohort compared to the AB0c cohort (14% vs. 29%). These included a range of viral infections, including EBV, gastrointestinal viruses, and SARS‐CoV‐2, as well as febrile infections like pyelonephritis and pneumonia. Neither group exhibited any instances of wound infection. Furthermore, at the 36‐month post‐KTx mark, the incidence of infections, particularly pneumonia and urinary tract infections, was higher in the AB0c cohort compared to the AB0i group. Six infectious episodes in five patients (50%) of the AB0i group had been documented within 36 months after KTx. During the same time, we noted 55 episodes in 22 patients (73%) in the AB0c group.

No cases of bleeding complications were observed in our complete cohort of transplant recipients.

None of the 10 patients of the AB0i group experienced rejection episodes related to AB0 antibody rebound. We did not observe any late reappearance of isoagglutinin antibodies during the entire follow‐up period. B cells were checked 4–6 weeks after transplantation so that a second dose of rituximab could be administered in the event of an unexpectedly rapid recurrence of B cells. No patient required further apheresis treatment or another dose of rituximab during the entire observation period; no B cells were found in any patients within the first 6 months after transplantation.

Rejections occurred earlier in the AB0c group. Median time to the first rejection was 12 (1–36) months, while in the AB0i group we observed the first rejection after 24 with a median of 27 (24–36) months. In the AB0i group, we observed three rejection episodes in three of 10 patients (one antibody mediated rejection (ABMR), Banff IIB, and two acute T‐cell mediated rejections, Banff Borderline and IA) within the first 36 months after KTx. ABMR was treated with rituximab 375 mg/m^2^ BSA and intravenous immunoglobulins. One patient required additional treatment with IA. In the AB0c group, we observed seven episodes of biopsy‐proven rejection in six patients during the first 36 months, one of which was an ABMR (3%) with Banff classification IIB. All others were acute T‐cell mediated rejections (4× Borderline Banff classification, one Banff IA, one IIA), all of which were well controlled with methylprednisone pulse therapy. After the rejection episodes, eGFR decreased to a new baseline of −2 mL/min/1.73 m^2^ BSA (±2.39).

### 3.5 Patient and Graft Survival After KTx

**FIGURE 2:**
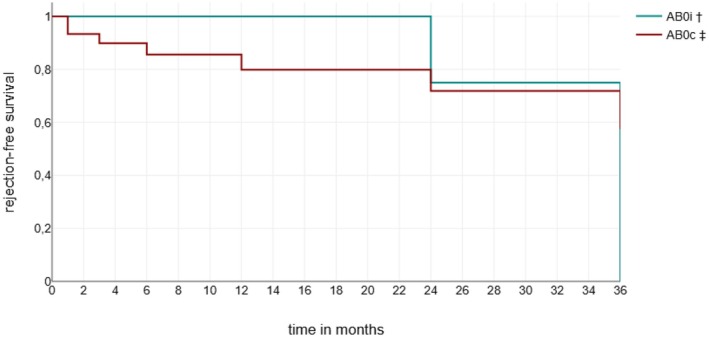
Time until any rejection for group AB0i and AB0c for 36 months after KTx. ^†^AB0i AB0 incompatible transplantation, ^‡^AB0c AB0 compatible transplantation.

There was no statistical difference between the AB0i and AB0c overall 3‐year patient survival rates (*p* = 0.24) and graft survival rates (*p* = 0.08) (see Figure 2).

In the AB0i group, the 3‐year graft survival rate was 100% after KTx. In the AB0c KTx group, the 3‐year graft survival was 93.3%. Graft loss occurred in 2 AB0c patients. The causes of graft loss were recurrence of IgA nephropathy after 24 months after KTx in one patient and 36 months after KTx in the other.

Patients' 3‐year survival rates were 100% in the AB0i and 96.6% in the AB0c group after KTx. The first patient in the AB0c died after 9 months, the second died after our main observation period, after 69 months. Table 6 provides an overview of patient and graft survival and patient loss to follow up at different points in time.

**TABLE 6:** Graft and Patient survival in AB0i and AB0c over different observation periods.

	AB0i group				AB0c group			
	*n*	Loss to follow‐up	Graft loss	Patient death	*n*	Loss to follow‐up	Graft loss	Patient death
KTx	10				30			
1 year	10	0	0	0	28	1	0	1
2 years	9	1	0	0	26	1	1	0
3 years	7	2	0	0	20	5	1	0

Abbreviations: AB0c KTx, AB0 compatible kidney transplantation; AB0i KTx, AB0 incompatible kidney transplantation.

Both patients had PTLD, one of which occurred after 6 months and led to the patient's death after 9 months. At the time of diagnosis, the patient had a Vasudev score of 13.8 that was higher than the median score of 11.0 in this group. The patient was in the high‐risk group for EBV infection with a recipient EBV‐negative, donor EBV‐positive constellation at the time of AB0c KTx. We observed first EBV copies 4 months after KTx and a maximum of 9470 copies 7 months after KTx. The second case of PTLD in the AB0c group occurred for the first time 10 months after KTx and relapsed 55 months later, resulting in the child's death 69 months after KTx. The 2nd patient had a Vasudev score of 11.7 at the time of diagnosis and was thus almost in the range of the median of the AB0c children of 11.0. The EBV constellation of this patient at the time of KTx had been unclear; the recipient had been EBV negative. The first EBV copies were detected in the patient 2 months after KTx and increased to a maximum of 230 000 copies by the time of PTLD diagnosis 6 months after KTx. After diagnosis, the Vasudev score was reduced to 7.15 in patient 1 and 4.44 in patient 2.

## 4 Discussion

To address the shortage of available transplant kidneys, protocols have been developed to allow AB0i living donor KTx. Between 2012 and 2024, 10 AB0i KTx were performed in pediatric recipients at our center. Patient and graft survival and complication rates were compared with those of 30 AB0‐compatible pediatric recipients matched for sex, height, and weight at the time of KTx. The results showed that midterm graft survival in recipients of AB0i transplants was comparable to the results of AB0‐compatible transplants. Our results do not differ from data in adult patients [14].

### 4.1 Complications

#### 4.1.1 Rejections

Recipients of an AB0i living‐donor kidney transplant who have undergone reduction of AB0 antibodies and B‐cell depletion do not show worse graft or patient survival rates when compared to those who have undergone an AB0c transplant [19]. Nonetheless, in literature ABMR represents the most frequent cause of graft loss in AB0i transplantation [20]. The risk of ABMR is associated with the isoagglutinin levels present at the time of transplantation and the presence of anti‐HLA antibodies [14].

The incidence of ABMR in AB0i kidney transplantation is estimated to be between 10% and 30% [20]. In the meta‐analysis conducted by Lo et al. [21], the incidence of acute rejection was found to be 32.9%, with the majority of cases being ABMR. ABMR is most prevalent in the initial posttransplant period, typically within the first 2 weeks [22]. In our AB0i observation group, we have not seen such early rejections, but we have seen a similar number of ABMR. In total, we observed three rejection episodes in three of 10 patients within the first 36 months after Ktx and thus have a similar occurrence to that reported in the literature. In the AB0c group, the rate of rejections in the first 36 months was lower. One ABMR in one patient and 6 episodes of acute T‐cell rejections in five patients could be observed. It might be expected that B‐cell depletion by rituximab administration would provide some protection against the development of donor specific antibodies (DSA). This appears to be the case in the first few months, but does not have a positive effect over a follow‐up period of > 12 months. Although we see a higher number of rejections in the AB0i group within the first 36 months, the GFR course and the overall graft survival is not worse during this period (see Figure 1).

#### 4.1.2 Infections

The prevalence of infectious complications varies between studies [10, 23]. It is likely that the discrepancy is due to the intensity of the desensitization strategies, which include the number and type of apheresis treatments, as well as the doses of immunosuppressants. A review of the literature using adult data, as there is no corresponding pediatric data, shows that AB0i recipients have a higher incidence of wound infection, pneumonia, and urinary tract infection or pyelonephritis in the first 90 days after transplantation compared with AB0‐compatible recipients [24, 25, 26]. In adjusted models, AB0 incompatibility was associated with a 2‐fold increased risk of pneumonia and a 56% increased risk of urinary tract infection or pyelonephritis in the first 90 days after transplantation, as well as a 3.5‐fold increased risk of wound infection between days 91 and 365. A2‐incompatible transplantation was associated with an increased risk of urinary tract infection or pyelonephritis only in the early posttransplant period [24]. In our study group, infectious complications within the first 90 days following KTx were lower in the AB0i cohort compared to the AB0c cohort (14% vs. 29%). This discrepancy may be attributed to the higher prevalence of urinary tract obstruction and pulmonary hypoplasia among the AB0c cohort (22% AB0i, 40% AB0c). Interestingly, exposure to immunosuppressive drugs was even slightly higher in the AB0i cohort. This is probably due to the fact that all patients in the AB0i group received weight‐adjusted induction with basiliximab, while only a few patients (known for poor compliance in the past, 2nd KTx) in the AB0c group received basiliximab. This leads to stronger immunosuppression in the AB0i group. The additional immunosuppression caused by the administration of rituximab and therapeutic apheresis was not detrimental to patient and organ survival and function at the time of observation or to overall patient health.

## 5 Conclusions

We conclude that AB0 incompatible kidney transplants can be performed with excellent results in children and adolescents after B cell depletion, isoagglutinin removal and induction with basiliximab.

Our midterm outcome of AB0i KTx is not inferior to that of AB0c KTx. The incidence of rejection episodes was higher after AB0 incompatible KTx during the first 36 months, but graft survival and graft function were comparably good in both groups. There was no increase in infectious complications or malignancies with AB0i KTx despite the more intense immunosuppression. Our data provide further evidence that AB0i KTx with living donor is a safe, successful, and reasonable option to alleviate the organ shortage, also in a pediatric cohort.

## Author Contributions

C.T., S.I.M., L.T.W., D.L.S., and J.T.: conceptualization. C.T., L.T.W., and J.T.: methodology. C.T. and J.T.: validation. C.T. and J.T.: formal analysis and visualization. C.T., L.T.W., and J.T.: writing – original draft preparation. C.T., L.T.W., D.L.S., and J.T.: writing – review and editing. C.T., L.T.W., and J.T.: supervision. All authors contributed to the article and approved the submitted version.

## Funding

The authors have nothing to report.

## Disclosure

The authors have nothing to report.

## Supporting information


**Appendix S1:** petr70248‐sup‐0001‐AppendixS1.docx.

## Data Availability

The data that support the findings of this study are available on request from the corresponding author. The data are not publicly available due to privacy or ethical restrictions.
