# Supplementary material for: Midterm Outcome of AB0 Incompatible Kidney Transplantation in Children and Adolescents—A Single Center Experience
Source: Pediatr Transplant. 2026 Jan 22;30(1):e70248. doi: 10.1111/petr.70248 (PMC12825340; doi:10.1111/petr.70248)
Supplement: Supplementary file 1 — Appendix S1: petr70248‐sup‐0001‐AppendixS1.docx. [file PETR-30-e70248-s001.docx]

Supplement

Table 1 Virusstatus of all patients pre transplantationem

| **AB0c §** | **Pat** |  | **Pat** | **AB0i †** | **Pat** |  | **Pat** |
| --- | --- | --- | --- | --- | --- | --- | --- |
|  |  |  |  |  |  |  |  |
| ¥CMV πD- /‡‡R- | 10 | ††EBV D- / R- | 4 | CMV D- / R- | 5 | EBV D- / R- | 0 |
| CMV D- / R+ | 1 | EBV D- / R+ | 0 | CMV D- / R+ | 0 | EBV D- / R+ | 0 |
| CMV D+ / R- | 6 | EBV D+ / R- | 14 | CMV D+ / R- | 3 | EBV D+ / R- | 4 |
| CMV D+ / R+ | 13 | EBV D+ / R+ | 7 | CMV D+ / R+ | 2 | EBV D+ / R+ | 4 |
|  |  | EBV D unknown / R- | 2 |  |  | EBV D unknown / R- | 1 |
|  |  | EBV D unknown / R+ | 3 |  |  | EBV D unknown / R+ | 1 |

§ AB0c compatible; † AB0i AB0 incompatible; ¥ Cytomegalovirus; π Donor; ‡‡ Recipient; †† Epstein Barr Virus
